# The predictive values of admission characteristics for 28-day all-cause mortality in septic patients with diabetes mellitus: a study from the MIMIC database

**DOI:** 10.3389/fendo.2023.1237866

**Published:** 2023-08-01

**Authors:** Chengyu Yang, Yu Jiang, Cailin Zhang, Yu Min, Xin Huang

**Affiliations:** ^1^ West China School of Public Health and West China Fourth Hospital, Sichuan University, Chengdu, Sichuan, China; ^2^ Department of Cardiology, Chinese People's Liberation Army of China (PLA) Medical School, Beijing, China; ^3^ Department of Biotherapy and National Clinical Research Center for Geriatrics, Cancer Center, West China Hospital, Sichuan University, Chengdu, Sichuan, China

**Keywords:** sepsis, diabetes mellitus, glycosylated hemoglobin, intensive care unit, all-cause mortality

## Abstract

**Background:**

Septic patients with diabetes mellitus (DM) are more venerable to subsequent complications and the resultant increase in associated mortality. Therefore, it is important to make tailored clinical decisions for this subpopulation at admission.

**Method:**

Data from large-scale real-world databases named the Medical Information Mart for Intensive Care Database (MIMIC) were reviewed. The least absolute selection and shrinkage operator (LASSO) was performed with 10 times cross-validation methods to select the optimal prognostic factors. Multivariate COX regression analysis was conducted to identify the independent prognostic factors and nomogram construction. The nomogram was internally validated *via* the bootstrapping method and externally validated by the MIMIC III database with receiver operating characteristic (ROC), calibration curves, decision curve analysis (DCA), and Kaplan-Meier curves for robustness check.

**Results:**

A total of 3,291 septic patients with DM were included in this study, 2,227 in the MIMIC IV database and 1,064 in the MIMIC III database, respectively. In the training cohort, the 28-day all-cause mortality rate is 23.9% septic patients with DM. The multivariate Cox regression analysis reveals age (hazard ratio (HR)=1.023, 95%CI: 1.016-1.031, p<0.001), respiratory failure (HR=1.872, 95%CI: 1.554-2.254, p<0.001), Sequential Organ Failure Assessment score (HR=1.056, 95%CI: 1.018-1.094, p=0.004); base excess (HR=0.980, 95%CI: 0.967-0.992, p=0.002), anion gap (HR=1.100, 95%CI: 1.080-1.120, p<0.001), albumin (HR=0.679, 95%CI: 0.574-0.802, p<0.001), international normalized ratio (HR=1.087, 95%CI: 1.027-1.150, p=0.004), red cell distribution width (HR=1.056, 95%CI: 1.021-1.092, p=0.001), temperature (HR=0.857, 95%CI: 0.789-0.932, p<0.001), and glycosylated hemoglobin (HR=1.358, 95%CI: 1.320-1.401, p<0.001) at admission are independent prognostic factors for 28-day all-cause mortality of septic patients with DM. The established nomogram shows satisfied accuracy and clinical utility with AUCs of 0.870 in the internal validation and 0.830 in the external validation cohort as well as 0.820 in the septic shock subpopulation, which is superior to the predictive value of the single SOFA score.

**Conclusion:**

Our results suggest that admission characteristics show an optimal prediction value for short-term mortality in septic patients with DM. The established model can support intensive care unit physicians in making better initial clinical decisions for this subpopulation.

## Introduction

Sepsis is one of the leading life-threatening conditions caused by the dysregulated host response to infection (1–4). Due to the high incidence and subsequently, mortality risk, sepsis, and septic shock are the major medical problems, which affect millions of critically ill populations (3, 5). Sepsis is frequently observed in aging and cancer as well as immunosuppressive subpopulations (6). Despite recent improvements in diagnosis and treatment (including the use of organ support, antibiotics, and fluid resuscitation), sepsis remains a high hospitalization cost and fatal disease around the world (4, 7). How to make more precise clinical management decisions for septic patients with different comorbidities has raised wide concerns (8, 9).

Notably, diabetes mellitus (DM) is one of the most frequent comorbidities in critically ill patients (10). Globally, the prevalence of DM has quadrupled since the last 80s, which has become the ninth major cause of death (11, 12). The International Diabetes Federation (IDF) suggested that the number of DM would rise to almost 650 million by 2040 (11). To date, compelling evidence showed that patients with DM suffered from an increased risk of various infections (13–15) and represented the predominant population experiencing post-sepsis complications (16, 17). Moreover, septic patients with DM presented worse clinical outcomes during the hospitalization (10, 13, 17). The altered immune response and the hyperglycemia condition further assist the growth of microorganisms, which could lead to a more tough situation in septic patients with DM (15, 18). Therefore, identifying possible strategies to reduce in-hospital mortality and subsequent morbidity in such high-risk subpopulations would bring considerable clinical and social benefits. Several studies have explored the interaction between the blood glucose level or glycosylated hemoglobin or DM and sepsis (19–21). However, the results were inconsistent. Meanwhile, there was a persistent lack of studies on the evaluation of independent prognostic factors and models to guide the clinical decisions on septic patients with DM at initial admission.

To address the research gaps, we aim to determine the significant prognostic factors in septic patients with DM, based on a large-scale intensive care database involving hospitalized patients between 2001 and 2019. Furthermore, we aim to further establish and validate a feasible individualized model to assess ICU physicians to predict short-term mortality in septic patients with DM.

## Materials and methods

### Database

Two cohorts of septic patients admitted to the ICU coexisted with DM, from a publicly available real-world clinical database named Medical Information Mart for Intensive Care Database III (MIMIC III, version 1.4) and IV (MIMIC IV, version 2.2), and maintained by Beth Israel Deaconess Medical Center in Boston, MA, USA from 2001 to 2019 was included (22). The description of this large-scale database has been published in the previous literature in chapters (23–26). The first author was permitted to extract data from the database after passing the related examination. The reporting of this study followed the Strengthening the Reporting of Observational Studies in Epidemiology (STROBE) guidelines (27).

### Ethical approval

The MIMIC-III and MIMIC-IV databases used in our study were approved by the Institutional Review Boards (IRB) of the Massachusetts Institute of Technology and do not contain protected health information. As the clinical data were extracted from the MIMIC-III and MIMIC-IV databases, they did not contain any individually identifiable information. Informed consent and ethical approval were not required from the Ethics Committee of the West China Fourth Hospital.

### Study population

**Figure 1:**
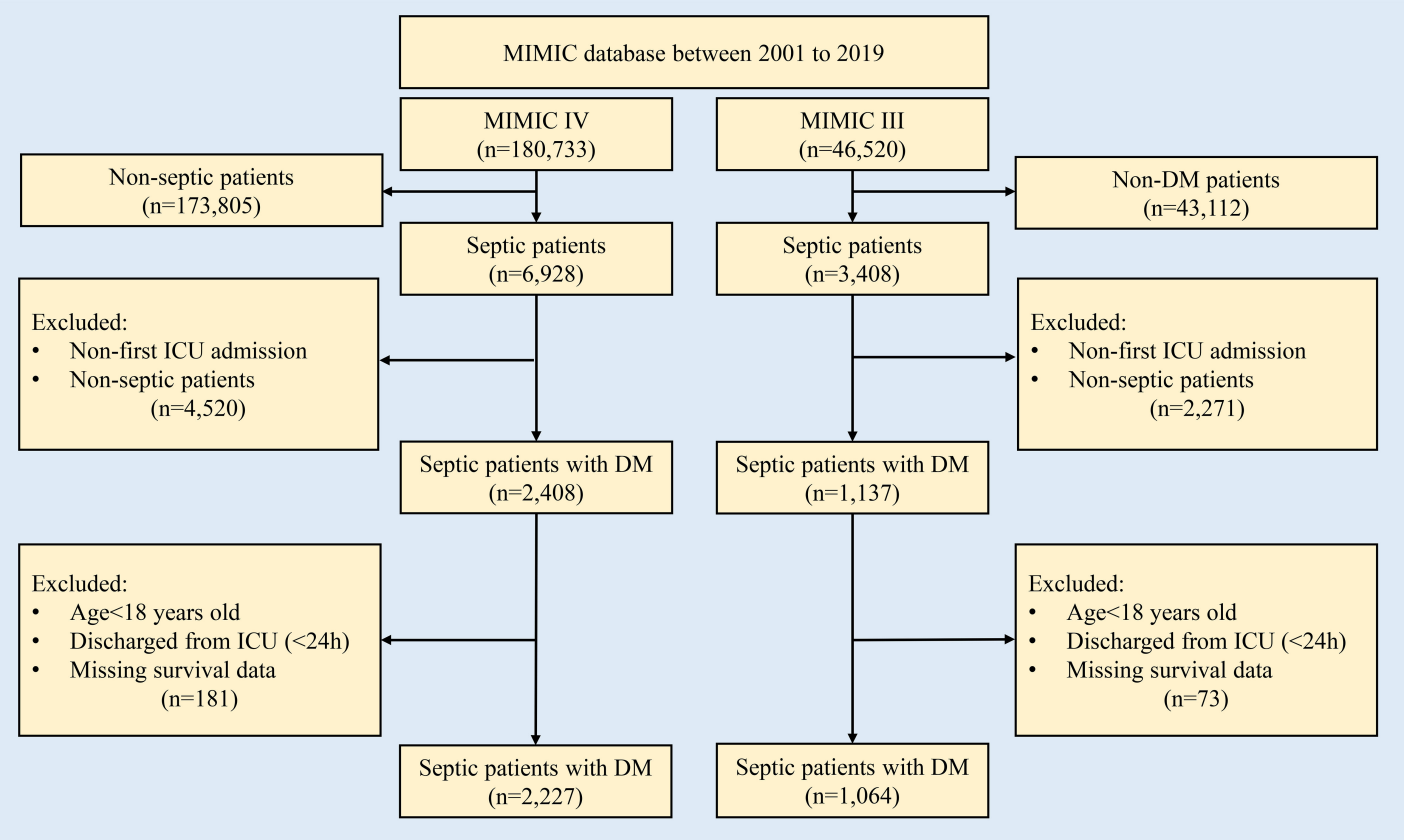
Flow chart for patient selection in this study. DM, diabetes mellitus; MIMIC, Medical Information Mart for Intensive Care Database.

Initially, the study population was focused on septic patients with DM. Additionally, the medical records of all adult patients aged at least 18 years admitted to the ICU were analyzed. To reduce selection bias, we only use first-round ICU admission records for patients who were enrolled in the ICU more than once. Patients who were discharged <24 hours and encountered missing variable data (medication information), as well as outcome data (28-day in-hospital mortality), were excluded. The flow chart of the patient selection process is shown in **Figure 1**.

### Definition of sepsis, septic shock, and DM

Sepsis is defined as life-threatening organ dysfunction caused by a dysregulated host response to infection (3). The diagnoses of sepsis and septic shock were based on the ‘Third International Consensus Definitions for Sepsis and Septic Shock (sepsis-3)’ (3). The organ dysfunction can be identified as an acute change in total SOFA score ≥2 points consequent to the infection. The SQL language method and the dictionary of codes for the International Classification of Diseases and Ninth Revision (ICD-9) and International Classification of Diseases and Tenth Revision (ICD-10) codes dictionary were used to screen and extract septic and septic shock patients with DM from the MIMIC III and MIMIC IV databases, respectively.

### Definition and selection of variables

The clinical characteristics of each patient were collected after admission to the ICU. Variables were divided into categorical and continuous variables. Categorical variables included sex (female and male), race (white, black, and other), weight, comorbidities (heart failure, respiratory failure, liver disease including hepatitis and cirrhosis, renal diseases including chronic kidney disease (CKD) and acute kidney injury (AKI), and malignancy), the medical usage records of continuous renal replacement therapy (CRRT), oxygen, ventilation, antibiotic, dopa, norepinephrine, and fluid input during the ICU stay.

Continuous variables included the age at admission, SOFA score, length of hospital stay, length of ICU stay, total fluids administrations, peripheral blood test data (red blood cell (RBC), platelets (PTL), white blood cell (WBC), albumin (Alb), alanine transaminase (ALT), glutamic transaminase (AST), hemoglobin (Hb), lactate (Lac), lymphocyte, neutrophil, international normalized ratio (INR), blood urea nitrogen (BUN), creatinine, potassium ion (K), chloride ion (Cl), sodium ion (Na), bicarbonate (HCO3^-^), prothrombin time (PT), mean erythrocyte volume (MCV), hematocrit (HCT), red cell distribution width (RDW)), arterial blood gas analysis (base excess (BE), total calcium (TCa), anion gap (AG)), glycosylated hemoglobin (HbA1c), fasting glucose, and general examination (heart rate (HR), mean blood pressure (MBP), temperature, respiration rate). There were thirty-eight variables at admission were selected for further analysis. The unit of the selected variable was summarized in **Table S1**.

### Primary outcome

The primary endpoint of this study was the 28-day all-cause mortality of septic patients with DM.

### Model construction and validation

Nomograms are widely used as clinical prognostic models in different fields of diseases (28). It can generate an individual probability of a clinical event by integrating diverse prognostic and determinant variables (29, 30). In this study, the patients from the MIMIC IV database were used for nomogram construction due to the larger sample size. Bootstrap analysis was used for internal validation. Moreover, patients from the MIMIC III database were further settled as the validation cohort.

### Statistical analysis

The variables with a missing value (<20%) were filled out using the median interpolation method (31). Categorical variables were presented as the number of the subgroup population with the percentage (%). Continuous variables were expressed as the mean and standard deviation (*Mean ± SD*). LASSO regression was used to remove less important variables and reduce the potential overfitting between included variables *via* the regression coefficients penalizing the size of the parameters. The LASSO regression curtailed the coefficient estimates toward zero, with the degree of shrinkage dependent on an additional parameter (λ) (32). To determine the optimal values for λ, 10-time cross-validation was used, and the minimum criteria λ was selected for further analysis (32). The multivariate Cox regression analysis was conducted to determine the independent short-term prognostic factors in septic patients with DM. The comparisons of selected variables among survivor and non-survivor subpopulations were conducted by using the Pearson-Chi square test or Student’s t-text as appropriate. The LASSO and stepwise multivariate Cox regression analyses were conducted by using the “rms” package from the “R” software (http://www.r-project.org, R Foundation, Vienna, Austria, version 4.1.2). The nomogram of prognostic factors associated with 28-day mortality in septic patients with DM was based on the statistically significant different variables during the multivariate Cox regression analysis.

The C-index (33) and the area under the receiver operating characteristic (ROC) curves (AUCs) were calculated for evaluating the discrimination of the nomogram. Furthermore, the calibration curves and decision curve analysis (DCA), and Kaplan-Meier (KM) curves were performed to assess the utility of the nomogram. A two-tailed *P-*value < 0.05 was considered statistically significant.

## Results

### The clinical features of septic patients with DM

In this study, 2,227 patients in the MIMIC IV database and 1,064 in the MIMIC III database who met the inclusion criteria were enrolled. In the training data, the overall 28-day all-cause mortality rate of septic patients with DM was 23.9% (n=533) and most of the patients were white in race (61.1%) and the male subpopulation showed a relatively higher proportion (58.3%). The septic shock was identified in 57.6% of septic patients from the MIMIC IV database and 51.2% of septic patients from the MIMIC III database, respectively. Septic patients with DM were accompanied by various degrees of comorbidities at admission, with a more pronounced in renal diseases (82.4%), respiratory failure (46.9%), and heart failure (39.6%) in the training cohort. During ICU hospitalization, the majority of the septic patients with DM received ventilation (83.0%) and antibiotic (98.5%) therapeutic interventions but only 11.3% of the patients received CRRT management. In the external validation cohort from the MIMIC III database, several differences were noticed in the proportions of heart and liver comorbidities, ventilation intervention, dopa usage, total fluids administrations, and length of ICU stay, compared with the training cohort (p<0.001). In addition, the mean proportion of HbA1c was 7.27% in the training cohort and 8.38% in the validation cohort, respectively. The clinical characteristics of the entire study population are shown in **Table 1**.

**Table 1:** The clinical characteristics of the whole study population.

Variable	Training cohort ^a^ (n=2,227)	Validation cohort ^b^ (n=1,064)	*P* ^c^
Baseline			
Age	67.98 ± 13.55 ^*^	69.09 ± 13.77 ^*^	**<0.001**
Race (%)			
White	1,361 (61.1)	729 (68.5)	**<0.001**
Black	299 (13.4)	126 (11.8)	
other	567 (25.5)	209 (19.6)	
Sex (%)			
Female	928 (41.7)	442 (41.5)	0.944
male	1,299 (58.3)	622 (58.5)	
Weight	88.08 ± 27.01 ^*^	88.70 ± 27.90 ^*^	0.542
SOFA	3.36 ± 2.07 ^*^	7.04 ± 3.76 ^*^	**<0.001**
Comorbidity			
COPD (%)			
Yes	212 (9.5)	18 (1.7)	**<0.001**
No	2015 (90.5)	1046 (98.3)	
Respiratory failure (%)			
Yes	1045 (46.9)	468 (44.0)	**<0.001**
No	1182 (53.1)	596 (56.0)	
Hypertension (%)			
Yes	545 (24.5)	467 (43.9)	**<0.001**
No	1682 (75.5)	597 (56.1)	
Atrial fibrillation (%)			
Yes	774 (34.7)	708 (66.5)	**<0.001**
No	1453 (65.2)	356 (33.5)	
Heart Failure (%)			
Yes	882 (39.6)	429 (40.3)	0.704
No	1345 (60.4)	635 (59.7)	
Renal failure (%)			
Yes	1835 (82.4)	896 (84.2)	0.198
No	392 (17.6)	168 (15.7)	
CKD (%)			
Yes	814 (36.5)	290 (27.3)	**<0.001**
No	1413 (63.5)	774 (72.7)	
Malignancies			
Yes	573 (25.7)	220 (20.7)	**0.002**
No	1654 (74.3)	844 (79.3)	
Liver diseases			
Yes	645 (29.0)	139 (13.1)	**<0.001**
No	1582 (71.0)	925 (86.9)	
Intervention			
CRRT (%)			
Yes	252 (11.3)	98 (9.2)	0.067
No	1,975 (88.7)	966 (90.8)	
Ventilation (%)			
Yes	2,194 (98.5)	1004 (94.4)	**<0.001**
No	33 (1.5)	60 (5.6)	
Antibiotic (%)			
Yes	2,194 (98.5)	1004 (94.4)	**<0.001**
No	33 (1.5)	60 (5.6)	
Dopa (%)			
Yes	139 (6.2)	159 (14.9)	**<0.001**
No	2,088 (93.8)	905 (85.1)	
NE (%)			
Yes	1,156 (51.9)	568 (53.4)	0.428
No	1,071 (48.1)	496 (46.6)	
Total fluid input	49,846.84 ± 92,181.40	59,717.59 ± 111,866.20	**0.007**
Length of hospital stay	14.81 ± 16.37	15.42 ± 15.98	0.313
Length of ICU stay	5.48 ± 7.18	7.86 ± 10.36	**<0.001**
Blood test			
Alb	2.75 ± 0.51 ^*^	2.71 ± 0.52 ^*^	**0.045**
ALT	108.87 ± 379.29 ^*^	113.39 ± 383.04 ^*^	0.750
AST	158.73 ± 563.50 ^*^	220.47 ± 1,041.81 ^*^	**<0.001**
BE	-3.40 ± 5.71 ^*^	-3.62 ± 5.51 ^*^	0.744
Hb	10.21 ± 2.10 ^*^	10.39 ± 2.02	**0.024**
Lac	2.74 ± 2.47 ^*^	2.60 ± 2.21 ^*^	0.114
Lymphocyte	9.50 ± 9.41 ^*^	8.93 ± 8.70 ^*^	0.096
Neutrophil	79.69 ± 12.95 ^*^	80.19 ± 13.39 ^*^	0.309
PCO_2_	41.54 ± 11.34 ^*^	41.03 ± 12.78 ^*^	0.250
PO_2_	95.23 ± 75.40 ^*^	123.81 ± 89.13 ^*^	**<0.001**
RBC	3.47 ± 0.74 ^*^	3.49 ± 0.70 ^*^	0.434
tCa	8.03 ± 0.88 ^*^	7.91 ± 0.91 ^*^	**<0.001**
AG	15.19 ± 5.01 ^*^	16.02 ± 4.60 ^*^	**<0.001**
INR	1.60 ± 0.87 ^*^	1.72 ± 1.08 ^*^	**<0.001**
BUN	39.12 ± 27.72 ^*^	39.49 ± 26.27 ^*^	0.715
WBC	15.35 ± 11.25 ^*^	15.31 ± 11.28 ^*^	0.910
Creatinine	2.10 ± 1.89 ^*^	2.08 ± 1.71 ^*^	0.696
K^+^	4.24 ± 0.85 ^*^	4.19 ± 0.81 ^*^	0.128
Cl^-^	103.28 ± 7.58 ^*^	105.54 ± 7.31 ^*^	**<0.001**
Na^+^	137.64 ± 6.28 ^*^	138.37 ± 6.05 ^*^	**0.002**
HCO_3_^-^	21.74 ± 11.99 ^*^	21.27 ± 5.32 ^*^	0.223
Pt	18.75 ± 12.58 ^*^	17.93 ± 9.28 ^*^	0.058
PLT	206.56 ± 120.49 ^*^	219.40 ± 125.29 ^*^	**0.005**
MCV	91.44 ± 7.88 ^*^	90.75 ± 7.63 ^*^	**0.018**
HCT	31.51 ± 6.24 ^*^	31.41 ± 6.01 ^*^	0.687
RDW	15.71 ± 2.40 ^*^	15.87 ± 2.29 ^*^	0.068
Glucose	190.06 ± 102.15 ^*^	178.70 ± 100.22 ^*^	**0.002**
HbA1c	7.27 ± 1.81 ^*^	8.38 ± 1.74 ^*^	**<0.001**
Admission condition			
Temperature	36.89 ± 0.95 ^*^	36.82 ± 0.83 ^*^	**0.039**
Respiration	21.35 ± 6.36 ^*^	20.60 ± 4.32 ^*^	**0.001**
HR	95.94 ± 20.95 ^*^	89.65 ± 16.52 ^*^	**<0.001**
MBP	78.82 ± 18.77 ^*^	72.25 ± 9.86 ^*^	**<0.001**
Septic shock			
No	944 (42.4)	519 (48.8)	**0.001**
Yes	1283 (57.6)	545 (51.2)	

a it contains the study population from the MIMIC IV database.

b it contains the study population from the MIMIC III database.

c Student’s t-test/Chi-square test.

^*^ Mean± standard deviation (SD).

COPD, chronic obstructive pulmonary disease; CKD, chronic kidney disease; CRRT, continuous renal replacement therapy; NE, norepinephrine; SOFA, Sequential Organ Failure Assessment; Alb, albumin; ALT, alanine transaminase AST, aspartate aminotransferase; BE, base excess; Hb, hemoglobulin; Lac, lactate; RBC, red blood cell; tCa, total calcium; AG, anion gap; INR, international normalized ratio; BUN, blood urea nitrogen; WBC, white blood cell; K^+^, potassium; Cl^-^, chlorine; Na, sodium; HCO_3_^-^, hydrocarbonate; Pt, prothrombin time; PLT, platelets; MCV, mean corpuscular volume; HCT, hematocrit; RDW, red blood cell distribution width; HbA1c, glycosylated hemoglobin; HR, heart rate; MBP, mean blood pressure. Bold value means statistically significant (p<0.05).

### LASSO regression analysis

**Figure 2:**
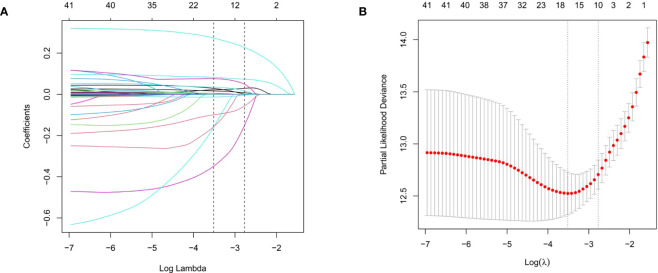
The LASSO regression analysis to select the potential variables. A total of thirty-eight variables are initially included and ten variables are finally selected for further analysis. **(A)** The LASSO coefficient analysis of the clinical features. **(B)** Tuning parameter selection in the LASSO Cox regression model.

A total of 38 variables were initially elected to the LASSO regression analysis with 10-fold cross-validation (**Figures 2A, B**; **Table S1**). Ultimately, ten variables including age, respiratory failure, SOFA score, Alb, BE, AG, INR, RDW, temperature, and HbA1c were selected for the stepwise multivariate Cox regression analysis (**Table S2**).

### The comparison of clinical variables between survivor and non-survivor

Based on the selected variables, we compared the clinical characteristics between the survivor and non-survivor subpopulations in 28-day all-cause mortality (**Table 2**). Regarding the age at admission, non-survivors were significantly older than the survivors (71.43 years *vs*. 66.89 years, p<0.001). Similarly, non-survivors showed a higher proportion of respiratory failure (691 cases (40.8%) *vs*. 354 (66.4%), p<0.001), SOFA score (4.08 *vs*. 3.13, p<0.001), RDW (16.53% *vs*. 15.45%, p<0.001), AG (18.44mEq/L *vs*. 14.17mEq/L, p<0.001) and prolonged INR (1.90s *vs*. 1.50s, p<0.001) as well as Hb1Ac (9.50% *vs*. 7.27%, p<0.001), when compared with the survivors. On the contrary, the non-survivors showed a decrease in Alb (2.67g/dL *vs*. 2.77g/dL, p<0.001), BE (-5.69mEq/L *vs*. -2.68mEq/L, p<0.001), and body temperature (36.61°C *vs*. 36.98°C, p<0.001) at admission, compared to the survivors. During the hospitalization, survivors showed longer hospitalization than non-survivors (length of hospital stay: 16.83 *vs*. 8.38 days, p<0.001; length of ICU stay: 5.57 *vs*. 5.21 days, p<0.001). There was a significant difference in total amounts of fluid input between survivors and non-survivors during the ICU stay (46720.13 ml *vs*. 59784.26 ml, p=0.004).

**Table 2:** Comparisons of clinical characteristics between survivors and non-survivors of the training cohort.

Variable	Subgroup	Survivors (n=1694)	Non-survivors (n=533)	*P*
**Age**	/	66.89 ± 13.56^*^	71.43 ± 12.92^*^	**<0.001**^a^
**Respiratory failure (%)**	Yes	691 (40.8)	354 (66.4)	**<0.001**^b^
	No	1003 (59.2)	179 (33.6)	
**SOFA score**	/	3.13 ± 1.82	4.08 ± 2.57	**<0.001**^a^
**BE**	/	-2.68 ± 4.88^*^	-5.69 ± 7.33^*^	**<0.001**^a^
**Alb**	/	2.77 ± 0.48^*^	2.67 ± 0.57 ^*^	**<0.001**^a^
**AG**	/	14.17 ± 3.88^*^	18.44 ± 6.58^*^	**<0.001**^a^
**INR**	/	1.50 ± 0.70^*^	1.90 ± 1.24^*^	**<0.001**^a^
**RDW**	/	15.45 ± 2.25^*^	16.53 ± 2.67^*^	**<0.001**^a^
**HbA1c**	/	7.27 ± 1.81^*^	9.50 ± 2.57^*^	**<0.001**^a^
**Length of hospital stay**	/	16.83 ± 17.89	8.38 ± 7.01	**<0.001**^a^
**Length of ICU stay**	/	5.57 ± 7.69	5.21 ± 5.24	**<0.001**^a^
**Total fluid input**	/	46,720.13 ± 95,902.51	59,784.26 ± 78,460.59	**0.004**
**Temperature**	/	36.98 ± 0.88^*^	36.61 ± 1.11^*^	**<0.001**^a^

^*^ Mean± standard deviation (SD).

a Student’s t-test.

b Pearson-Chi square test.

SOFA, Sequential Organ Failure Assessment; BE, base excess; Alb, albumin; AG, anion gap; ICU, intensive care unit; INR, international normalized ratio; RDW, red blood cell distribution width; HbA1c, glycosylated hemoglobin. Bold value means statistically significant (p<0.05).

### Multivariate Cox regression analyses

**Figure 3:**
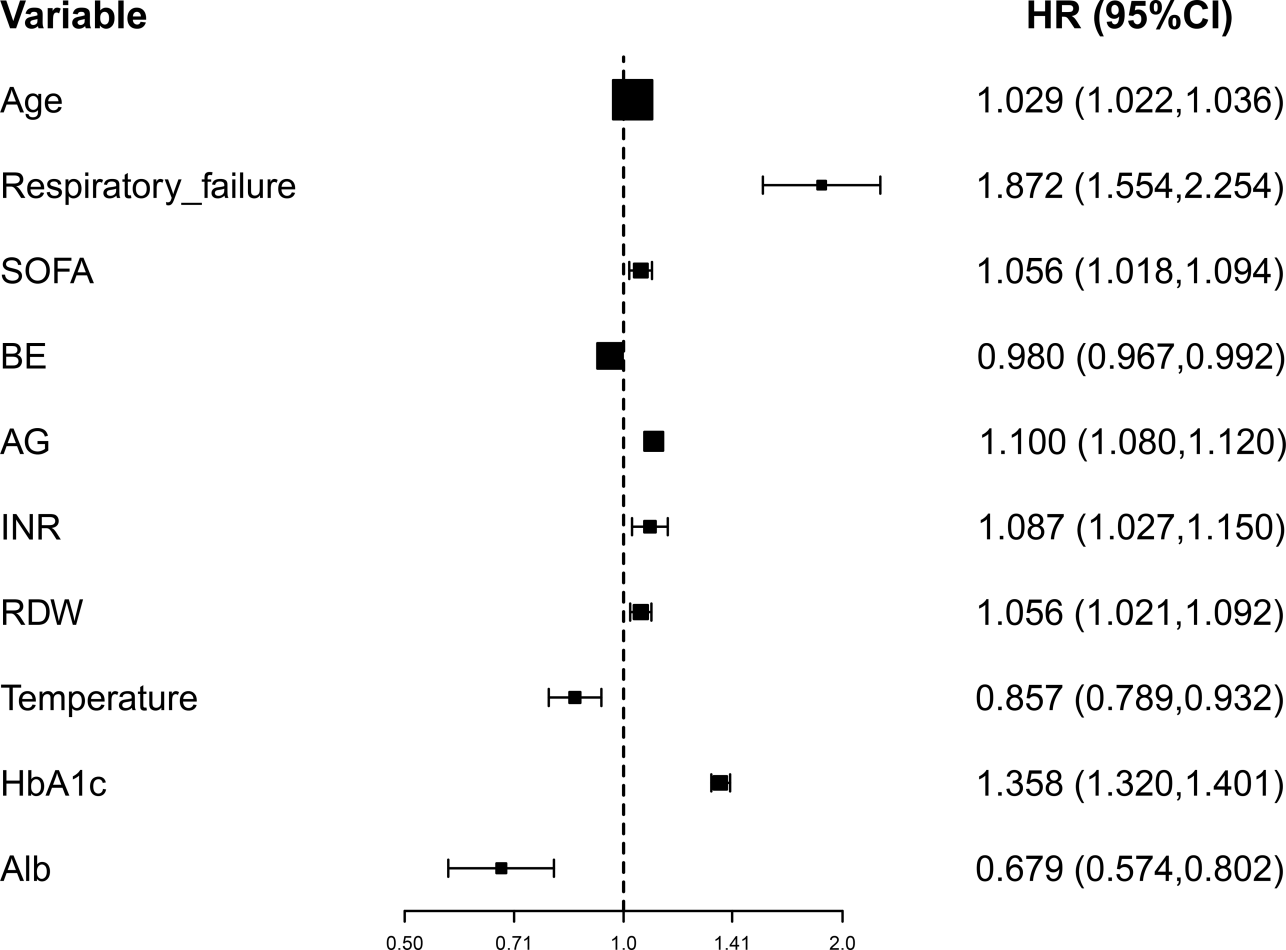
Forest plot for the multivariate Cox regression analysis. BE, base excess (mEq/L); SOFA, Sequential Organ Failure Assessment; AG, anion gap (mEq/L); INR, international normalized ratio (second); RDW, red blood cell distribution width (%), HbA1c, glycosylated hemoglobin (%).

Multivariate Cox regression analysis revealed that age at admission (hazard ratio (HR)=1.029 *per year*, 95%CI: 1.022-1.036, p<0.001), respiratory failure (HR=1.872, 95%CI: 1.554-2.254, p<0.001), SOFA (HR=1.056, 95%CI: 1.018-1.094, p=0.004), levels of BE (HR=0.980, 95%CI: 0.967-0.992, p=0.002), AG (HR=1.100, 95%CI: 1.080-1.120, p<0.001), Alb (HR=0.679, 95%CI: 0.574-0.802, p<0.001), INR (HR=1.087, 95%CI: 1.027-1.150, p=0.004), RDW (HR=1.056, 95%CI: 1.021-1.092, p=0.001), and body temperature (HR=0.857, 95%CI: 0.789-0.932, p<0.001), as well as HbA1c (HR=1.358, 95%CI: 1.320-1.401, p<0.001), were the independent prognostic factors in 28-day mortality of septic patients with DM (**Figure 3**).

### Nomogram construction and validation

**Figure 4:**
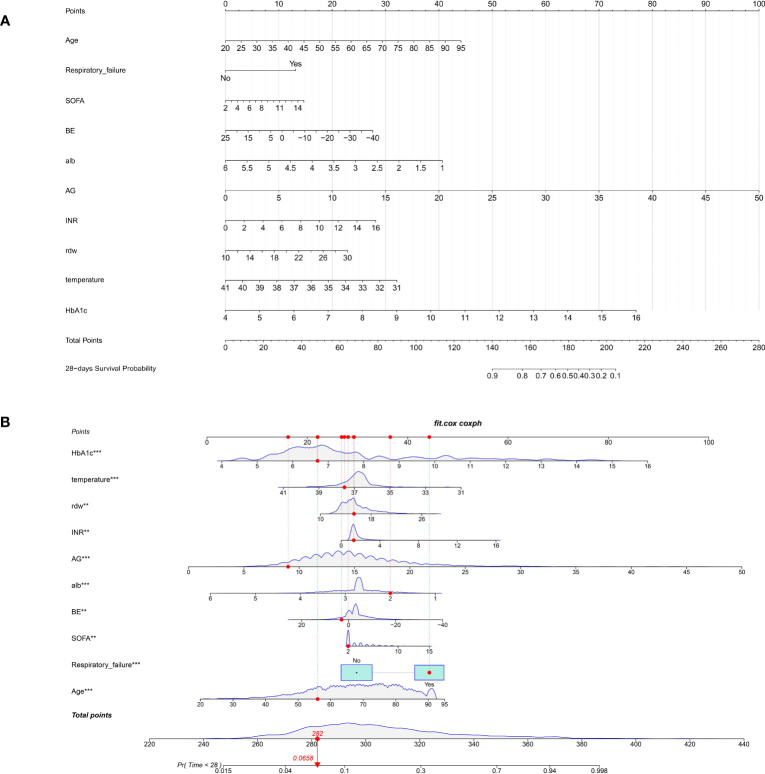
The nomogram for predicting the 28-day all-cause mortality in septic patients with diabetes mellitus. **(A)**. The nomogram is constructed by ten admission clinical variables; **(B)**. The example for explaining the clinical utility of the nomogram. The admitted patient would get an individualized score for each variable. By adding all scores, the clinicians could calculate the potential risk of 28-day all-cause mortality for each patient (red dot). BE, base excess (mEq/L); SOFA, Sequential Organ Failure Assessment; AG, anion gap (mEq/L); INR, international normalized ratio (second); RDW, red blood cell distribution width (%), HbA1c, glycosylated hemoglobin (%).

Depending on the prognostic factors determined by the multivariate Cox regression analysis, an individualized nomogram was subsequently established with the selected ten variables (**Figure 4A**). Each admitted septic patient with DM could get a total score and the corresponding risk for 28-day hospitalization mortality by adding the scores derived from the ten variables (**Figure 4B**).

**Figure 5:**
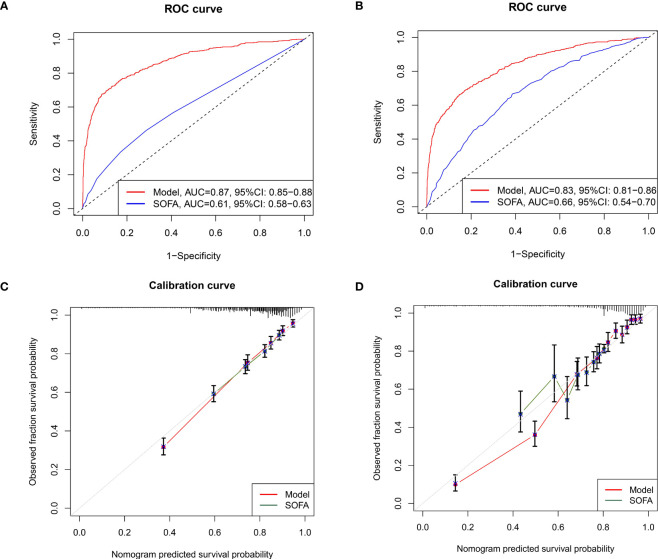
Methods to evaluate the accuracy and discrimination of the nomogram. **(A)** The ROC curves for evaluating the accuracy of the nomogram through internal bootstrapping analysis. **(B)** The ROC curves for evaluating the accuracy of the nomogram *via* external cohort analysis; **(C)** The calibration curves for evaluating the discrimination of the nomogram in the training cohort; **(D)** The calibration curves for evaluating the discrimination of the nomogram in the external validating cohort. ROC, receiver operating characteristic.

To validate the clinical utility of the nomogram, both internal bootstrap analysis and external cohort validation were performed. The C-indexes, which were consistent with the AUC of the ROCs, were all above 0.80, with 0.870 (95%CI: 0.850-0.880) in the internal bootstrap analysis and 0.830 (95%CI:0.810-0.860) in the external validation cohort, respectively (**Figures 5A, B**). Compared with the single SOFA score, our model was internally and externally validated to have better discrimination and accuracy in predicting the 28-day all-cause mortality of septic patients with DM (**Figures 5A, B**).

Besides, we applied the nomogram to the septic shock subpopulation to further validate the generalization of the model. The AUC of the ROC for predicting the 28-day mortality of the septic shock subpopulation reached 0.82 (95%CI: 0.790-0.850), which indicated the identified prognostic indicators were also pivotal in more severe subpopulations at admission (**Figure S1**).

### Robustness check

To further evaluate the utility of the nomogram, calibration curves were applied. Detailly, the calibration curves showed promising agreement in the ideal and observation events in both training (**Figure 5C**) and validation cohorts (**Figure 5D**) as well as the septic shock cohorts (**Figure S2**).

**Figure 6:**
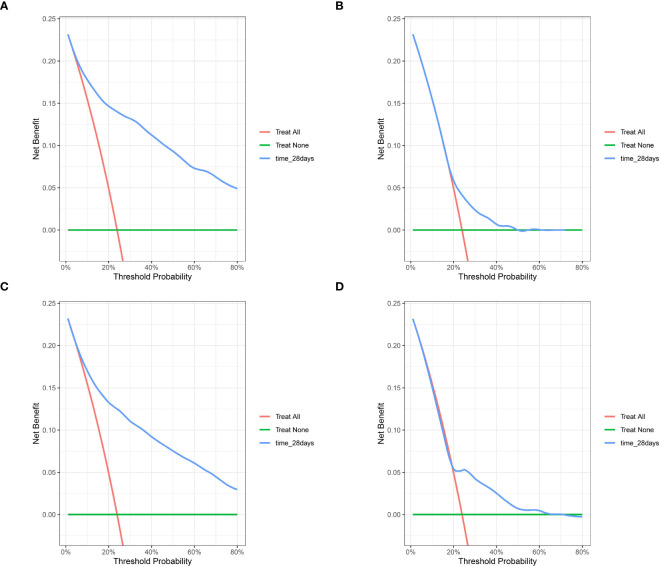
The DCA for evaluating the clinical utility of the nomogram. **(A)** the DCA of internal bootstrapping analysis; **(B)** The DCA of the training SOFA score; **(C)** The DCA of external validation cohort; **(D)** The DCA of the external SOFA score. DCA, decision curve analysis; SOFA, Sequential Organ Failure Assessment.

Similarly, the DCA curves showed that the scores derived from the nomogram could be more accurate than a treat-none or treat-all strategy when the threshold probability was above 5% in the training cohort (**Figure 6A**) and 10% in the septic shock subpopulation (**Figure S3**), respectively. Meanwhile, the external validation cohort also supported an optimal clinical utility of the model at the threshold probability intervals greater than 5% (**Figure 6C**), which was also better than the prediction value of SOFA scores (**Figures 6B, D**) in the study population.

**Figure 7:**
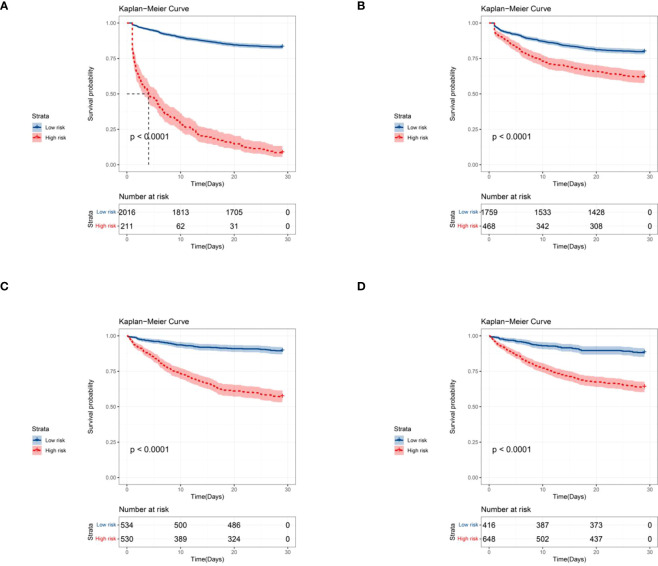
Kaplan-Meier curves for predicting the 28-day all-cause mortality in septic patients with DM by stratifying the different risk subgroups (the cut-off point was 186). **(A)** Kaplan-Meier curves based on the nomogram; **(B)** Kaplan-Meier curves based on the training SOFA score; **(C)** Kaplan-Meier curves based on the external validation cohort; **(D)** Kaplan-Meier curves based on the external validation SOFA score.

Based on the risk model, the KM curves showed good discrimination in identifying the real high-risk subpopulation (cutoff value: 186) in both the internal (**Figures 7A, B**) and external validating cohorts (**Figures 7C, D**).

## Discussion

Currently, prognostic factors for septic patients with DM remain limited. In this study, we fill this research gap and identify that various admission characteristics were significantly associated with the short-term mortality risk in septic patients with DM. Additionally, we developed an individualized prediction nomogram for the clinical management of septic patients with DM at the initial round evaluation. Robustness analyses showed good discrimination, calibration ability, and clinical usefulness of this predictive model.

Sepsis is still the leading cause of death in patients elected to the ICU (15, 18). In the present study, the 28-day mortality rate of septic patients with DM was 23.9% (n=533), which was higher than in general septic patients (10%-15%) (34–36). Regarding the hospitalization characteristics of the study population, we observed that survivors showed longer hospital and ICU stays than non-survivors, which was consistent with previous studies (24, 37). Besides, our study also determined that non-survivors received a higher volume of fluids input during the ICU stay than survivors. Interestingly, the optimal amount of fluid administration for septic and septic shock patients is still uncertain (24, 38–40). Notably, one meta-analysis included 13 trials that suggested that lower standard intravenous (IV) fluid volumes showed no significant difference in all-cause mortality compared with higher IV fluid volumes (41). Furthermore, the recent Conservative versus Liberal Approach to Fluid Therapy in Septic Shock in Intensive Care (CLASSIC) study also revealed that adult patients with septic shock might not get more survival or health-related quality of life benefits from restrictive fluid therapy when compared with IV fluid therapy (38). Thus, clinicians should trade off the risks and benefits of fluid administration in each stage of critical illness. Further works are needed to help clinicians to make tailored fluid management.

Among septic patients with DM, recent basic and clinical studies revealed that systemic immune dysfunctions including, but not limited to, deficiencies in neutrophil function and declining T-cell responses triggered by the hyperglycemia condition were associated with the infection development and sepsis mortality (10, 42). As one of the representative indicators in DM, HbA1c was mainly interpreted as a biomarker to reflect the short and intermediate terms of glycemic control. Of note, recent studies demonstrated the clinical predictive value of HbA1c in the development and prognosis of critically ill diseases, particularly sepsis. However, the findings were not always consistent. Notably, Anca et al. determined the nonlinear relationship between HbA1c and the risk of sepsis (43). However, HbA1c was not identified significantly associated with mortality in septic patients (43). On the contrary, the study by Guo et al. showed that the levels of HbA1c at admission were remarkably associated with morbidity and mortality in septic patients (44). In our analysis, high proportions of HbA1c were strongly associated with an increased risk of short-term mortality in septic patients with DM. Preclinical studies showed that chronic dysglycemia could impair the vascular endothelial glycocalyx and further cause complications and the mortality of sepsis (45). Especially, the lung was one of the most vulnerable organs in DM and septic patients. In our study, we observed that patients with respiratory failure showed a nearly two-fold risk (HR=1.872) of mortality, compared with patients without respiratory failure. Notably, several previous studies suggested that DM was associated with an increased risk of respiratory failure. For example, Gulcan et al. reported that half of the patients with DM exhibited respiratory failure during sleep, which might be due to restrictive impairment of respiratory function secondary to DM (46). Besides, the preclinical data also demonstrated that hyperglycemia could activate sodium-potassium-chloride cotransporter 1(NKCC1) related pathways and adversely affect alveolar fluid regulation and lung function (47). Therefore, septic patients with DM are expected to receive more active respiratory function surveillance during the ICU stay.

Compared with recent studies on general sepsis, we validated several clinical indicators in the subpopulation of patients with DM. Initially, we identified that age was a strong prognostic indicator of short-term mortality in septic patients with DM. There is a significant increase in the prevalence of DM in the elderly population (48–50), leading to exacerbation, poor prognosis, and increased mortality risk in sepsis (51). Additionally, serum biomarkers were important predictors in the surveillance of critically ill patients (34, 36, 52–55). Septic patients were often accompanied by hypoproteinemia due to capillary leakage, reperfusion injury, tissue ischemia, and inflammation (56, 57). More importantly, septic patients with hypoproteinemia showed worse clinical outcomes during hospitalization. Similarly, hypoproteinemia was also associated with multiple adverse complications and increased risk of adverse outcomes in DM (58). Therefore, septic patients with DM may suffer from a higher risk of mortality in the condition of hypoproteinemia. Furthermore, acid-base disorders are frequently observed in critically ill patients (59), especially in patients with DM (60). Of note, serum lac level was a tight biomarker for predicting the clinical outcome of patients with sepsis (61, 62). Interestingly, hyperlactatemia was a frequent metabolic condition in DM patients with ketoacidosis (63, 64). However, compared with other critically ill diseases, the elevated lac level was not determined to be associated with increased length of ICU stay or mortality in DM patients (65). Furthermore, some researchers even queried whether the standard hyperlactatemia cut-off value was also adapted for the survival prediction in DM patients (66). For these reasons, the value of lactate levels might be undermined in predicting short-term mortality in septic patients with DM, particularly when they were concurrent with diabetic ketoacidosis.

As a common biomarker for evaluating the acid-base balance, the AG levels were frequently used to evaluate the type of metabolic acidosis. However, acid-base imbalance, especially metabolic acidosis, frequently occurred in seriously ill patients and was significantly related to mortality. In our analysis, the levels of BE and AG were determined to be significantly associated with short-term mortality among septic patients with DM. Recent two studies from different regions also highlighted the predictive role of BE in the diagnosis and prognosis of sepsis (67, 68). Moreover, AG was recently identified to be an optimal serum biomarker for reflecting systemic dysfunctions in critically ill patients (69–71). In particular, high levels of AG were not only associated with impaired cardiorespiratory fitness (72) but also mediated more severe insulin resistance conditions (73). This could partially explain the underlying mechanism that AG played an important role in the prognosis of septic patients with DM. Currently, whether AG can accurately predict the prognosis of critically ill patients is still debatable. These discrepancies may be due to differences in varied study populations and measuring methods as well as other factors that influence AG values may also influence the exact correlations. Further studies are warranted to explore the underlying pathophysiological mechanism.

Consistent with the study by Liu et al. (74), we observed that prolonged INR was positively associated with short-term mortality in septic patients with DM. Some possible mechanisms might explain this finding. It is noteworthy that the activated coagulation system was regarded as the primary response of host systemic defense in sepsis (75). However, chronic septic conditions could adversely mediate the coagulation dysfunction with subsequent disseminated intravascular coagulation (DIC) (76). Besides, we also determined that a high percentage of RDW was an adverse prognostic factor for septic patients with DM. Most recently, the latest evidence showed that RDW could predict poor outcomes in various chronic diseases (77–81). In the DM population, Nada found that RDW was markedly higher in DM patients than in healthy subjects and particularly higher in uncontrolled glycemia (82). And in the population with sepsis, a meta-analysis of three studies revealed that a high level of RDW was associated with septic death (83). Thus, the percentage of RDW, affected by the hyperglycemia condition, showed a unique clinical value in critically ill patients, which could be a supplement indicator for assessing the prognosis of septic patients with DM.

Interestingly, the admitting body temperature was observed to be associated with the severity and prognosis of septic patients with DM. The latest two large-scale multi-center studies yielded the distinguished role of body temperature in the prognosis of critically ill patients, especially in terms of septic patients (84, 85). Similarly, our study supported that the patients with relatively higher body temperatures presented a lower risk for short-term mortality.

Based on identified prognostic factors, we established a new individualized nomogram to evaluate the condition of septic patients with DM at admission. The optimal C-index and AUCs in both the internal and external validation cohorts suggested the good discrimination and accuracy of the model in detecting high-risk patients. Furthermore, compared with the separate SOFA evaluation system, our model showed better predictive accuracy combined with various clinical characteristics. Additionally, we explored the utility of the nomogram in septic shock patients with DM, the subpopulation which was much more related to mortality. As expected, the model also presented promising predictive ability in detecting high-risk septic shock patients. Therefore, by using the predictive model, clinicians could better stratify risk for septic patients with DM at the initial evaluation.

Nevertheless, our study has some limitations. First, some factors, including laboratory indicators (such as fast plasma glucose, fasting insulin, fasting C-peptide, and C-reactive protein), site of infection, drugs, and interventions that could be related to prognosis, were not included in this study. Second, although this is a large-scale cohort study with over 3000 patients involved, the nature of the retrospective study design could inevitably lead to selection bias. Third, the MIMIC database was constructed from the medical records of admitted patients during the past two decades. However, intensive care medicine has developed substantially during the same period. Whether our findings would also be adapted to the current clinical practice needs further validation. Last, although we have successfully developed and validated a short-term mortality risk stratification model, data from other countries or regions are needed to verify this model in the future with more useful variables added.

## Conclusions

In this study, ten clinical variables including age, respiratory failure, SOFA, BE, Alb, AG, INR, RDW, HbA1c, and temperature at admission were identified as independent prognostic factors in predicting the 28-day all-cause mortality in septic patients with DM. Based on these factors, we developed and externally validated a predictive nomogram, with optimal discrimination and accuracy to detect the high-risk subgroup. This model can be implemented for ICU physicians to quickly make the initial clinical decision for septic patients with DM in clinical practice.

## Data availability statement

The original contributions presented in the study are included in the article/**Supplementary Material**. Further inquiries can be directed to the corresponding author.

## Author contributions

(I) Conception and design: XH and CY. (II) Administrative support: XH. (III) Provision of study materials or patients: CY, YJ, and YM. (IV) Collection and assembly of data: CY, YJ, and YM. (V) Data analysis and interpretation: XH, CY, and CZ. (VI) Manuscript writing: All authors. (VII) Final approval of manuscript: All authors.
