# Supplementary material for: The predictive values of admission characteristics for 28-day all-cause mortality in septic patients with diabetes mellitus: a study from the MIMIC database
Source: Front Endocrinol (Lausanne). 2023 Aug 1;14:1237866. doi: 10.3389/fendo.2023.1237866 (PMC10442168; doi:10.3389/fendo.2023.1237866)

**Supplementary Material**

**The Predictive Values of Admission Characteristics for 28-day All-cause Mortality in Septic Patients with Diabetes Mellitus: A Study from the MIMIC Database**

**Table S1.** The units of the continuous variables in this study.

| **Variable** | **Units** |
| --- | --- |
| **Baseline** | |
| **Age** | year |
| **Weight** | kg |
| **SOFA** | / |
| **MBP** | mmHg |
| **Temperature** | ℃ |
| **Respiratory rate** | bpm |
| **HR** | bpm |
| **Blood test** | |
| **Alb** | g/dL |
| **ALT** | IU/L |
| **AST** | IU/L |
| **BE** | mEq/L |
| **Hb** | g/dL |
| **Lac** | mmol/L |
| **Lymphocyte** | % |
| **Neutrophil** | % |
| **RBC** | m/uL |
| **tCa** | mg/dL |
| **AG** | mEq/L |
| **INR** | second |
| **BUN** | mg/dL |
| **WBC** | K/uL |
| **Creatinine** | mg/dL |
| **K^+^** | mEq/L |
| **Cl^-^** | mEq/L |
| **Na^+^** | mEq/L |
| **HCO_3_^-^** | mEq/L |
| **PT** | second |
| **PLT** | K/uL |
| **MCV** | fL |
| **HCT** | % |
| **RDW** | % |
| **Glucose** | mmol/L |
| **HbA1c** | % |

*Abbreviation: SOFA: Sequential Organ Failure Assessment; Alb: albumin; ALT: alanine transaminase AST: aspartate aminotransferase; BE: base excess; Hb: hemoglobulin; Lac: lactate; RBC: red blood cell; tCa: total calcium; AG: anion gap; INR: international normalized ratio; BUN: blood urea nitrogen; WBC: white blood cell; K^+^: potassium; Cl^-^: chlorine; Na^+^: sodium; HCO3-: hydrocarbonate; Pt: prothrombin time; PLT: platelets; MCV: mean corpuscular volume; HCT: hematocrit; RDW: red blood cell distribution width; HbA1c: glycosylated hemoglobin; HR: heart rate; MBP: mean blood pressure.*

**Table S2**. The multivariable Cox regression analysis of the selected variables in the LASSO model.

| **Variables** | **Coef.** | **S. E** | **z** | **HR** | **95%CI (low)** | **95%CI (up)** | ***P*** |
| --- | --- | --- | --- | --- | --- | --- | --- |
| Age | 0.028 | 0.004 | 7.903 | 1.0285 | 1.022 | 1.036 | **<0.001** |
| Respiratory failure | 0.627 | 0.095 | 6.585 | 1.872 | 1.554 | 2.254 | **<0.001** |
| SOFA | 0.054 | 0.018 | 2.901 | 1.056 | 1.018 | 1.094 | **0.004** |
| BE | -0.020 | 0.007 | -3.04 | 0.980 | 0.967 | 0.992 | **0.002** |
| AG | 0.095 | 0.007 | 13.355 | 1.100 | 1.080 | 1.120 | **<0.001** |
| INR | 0.083 | 0.029 | 2.878 | 1.087 | 1.027 | 1.150 | **0.004** |
| RDW | 0.054 | 0.017 | 3.173 | 1.056 | 1.021 | 1.092 | **0.001** |
| Temperature | -0.153 | 0.043 | -3.593 | 0.857 | 0.789 | 0.932 | **<0.001** |
| HbA1c | 0.306 | 0.016 | 19.357 | 1.358 | 1.320 | 1.401 | **<0.001** |
| Alb | -0.388 | 0.085 | -4.545 | 0.679 | 0.574 | 0.802 | **<0.001** |

*Abbreviation: SOFA: Sequential Organ Failure Assessment; BE: base excess; AG: anion gap; INR: international normalized ratio; RDW: red blood cell distribution width; HbA1c: glycosylated hemoglobin; Alb:* *albumin.*

**Table S3**. The demographic characteristics of the septic shock patients with diabetes mellitus.

| **Variables** | **Septic shock**  **(**n=1283**)** |
| --- | --- |
| **Age** | 68.22±13.25 |
| **Sex (%)** |  |
| **Female** | 541(42.2) |
| **male** | 742(57.8) |
| **SOFA** | 3.71±2.27 |
| **Respiratory failure (%)** |  |
| **Yes** | 699(54.5) |
| **No** | 584(45.5) |
| **Heart Failure (%)** |  |
| **Yes** | 522(40.7) |
| **No** | 761(59.3) |
| **Renal failure (%)** |  |
| **Yes** | 1094(85.3) |
| **No** | 189(14.7) |
| **Malignancies (%)** |  |
| **Yes** | 325(25.3) |
| **No** | 958(74.7) |
| **Liver diseases (%)** |  |
| **Yes** | 392(30.5) |
| **No** | 891(69.4) |
| **CRRT (%)** |  |
| **Yes** | 204(15.9) |
| **No** | 1079(84.1) |
| **Ventilation (%)** |  |
| **Yes** | 1116(87.0) |
| **No** | 167(13.0) |
| **Antibiotic (%)** |  |
| **Yes** | 1270(99.0) |
| **No** | 13(1.0) |
| **Dopa (%)** |  |
| **Yes** | 109(8.5) |
| **No** | 1174(91.5) |
| **NE (%)** |  |
| **Yes** | 942(73.4) |
| **No** | 341(26.6) |
| **Total fluid input** | 63,462.12±105,094.10 |
| **Length of hospital stay** | 14.83±15.27 |
| **Length of ICU stay** | 6.36±7.80 |
| **Alb** | 2.71±0.52 |
| **ALT** | 137.22±467.27 |
| **AST** | 203.92±672.14 |
| **BE** | -4.35±6.19 |
| **Hb** | 10.21±2.09 |
| **Lac** | 3.06±2.81 |
| **Lymphocyte** | 9.23±9.46 |
| **Neutrophil** | 79.84±13.12 |
| **PCO_2_** | 42.03±12.24 |
| **PO_2_** | 96.04±78.48 |
| **RBC** | 3.47±0.74 |
| **tCa** | 7.94±0.88 |
| **AG** | 15.76±5.56 |
| **INR** | 1.65±0.87 |
| **BUN** | 41.55±27.94 |
| **WBC** | 16.22±12.15 |
| **Creatinine** | 2.24±1.94 |
| **K^+^** | 4.29±0.87 |
| **Cl^-^** | 103.32±7.93 |
| **Na^+^** | 137.68±6.59 |
| **HCO_3_^-^** | 21.03±12.56 |
| **Pt** | 19.53±13.17 |
| **PLT** | 204.14±118.42 |
| **MCV** | 91.84±8.05 |
| **HCT** | 31.61±6.27 |
| **RDW** | 15.91±2.51 |
| **Glucose** | 194.04±110.98 |
| **HbA1c** | 7.90±2.31 |
| **Temperature** | 36.82±1.02 |
| **Respiration** | 21.62±6.55 |
| **HR** | 95.93±21.31 |
| **MBP** | 77.00±19.04 |
| **28-day mortality** | 396(30.8) |

Abbreviations: SOFA: Sequential Organ Failure Assessment; CRRT: continuous renal replacement therapy; NE: norepinephrine; Alb: albumin; ALT: alanine transaminase AST: aspartate aminotransferase; BE: base excess; Hb: hemoglobulin; Lac: lactate; RBC: red blood cell; tCa: total calcium; AG: anion gap; INR: international normalized ratio; BUN: blood urea nitrogen; WBC: white blood cell; K^+^: potassium; Cl^-^: chlorine; Na: sodium; HCO_3_^-^: hydrocarbonate; Pt: prothrombin time; PLT: platelets; MCV: mean corpuscular volume; HCT: hematocrit; RDW: red blood cell distribution width; HbA1c: glycosylated hemoglobin; HR: heart rate; MBP: mean blood pressure.

**Figure S1**. The ROC curve for evaluating the predictive accuracy of the nomogram in septic shock patients with DM.


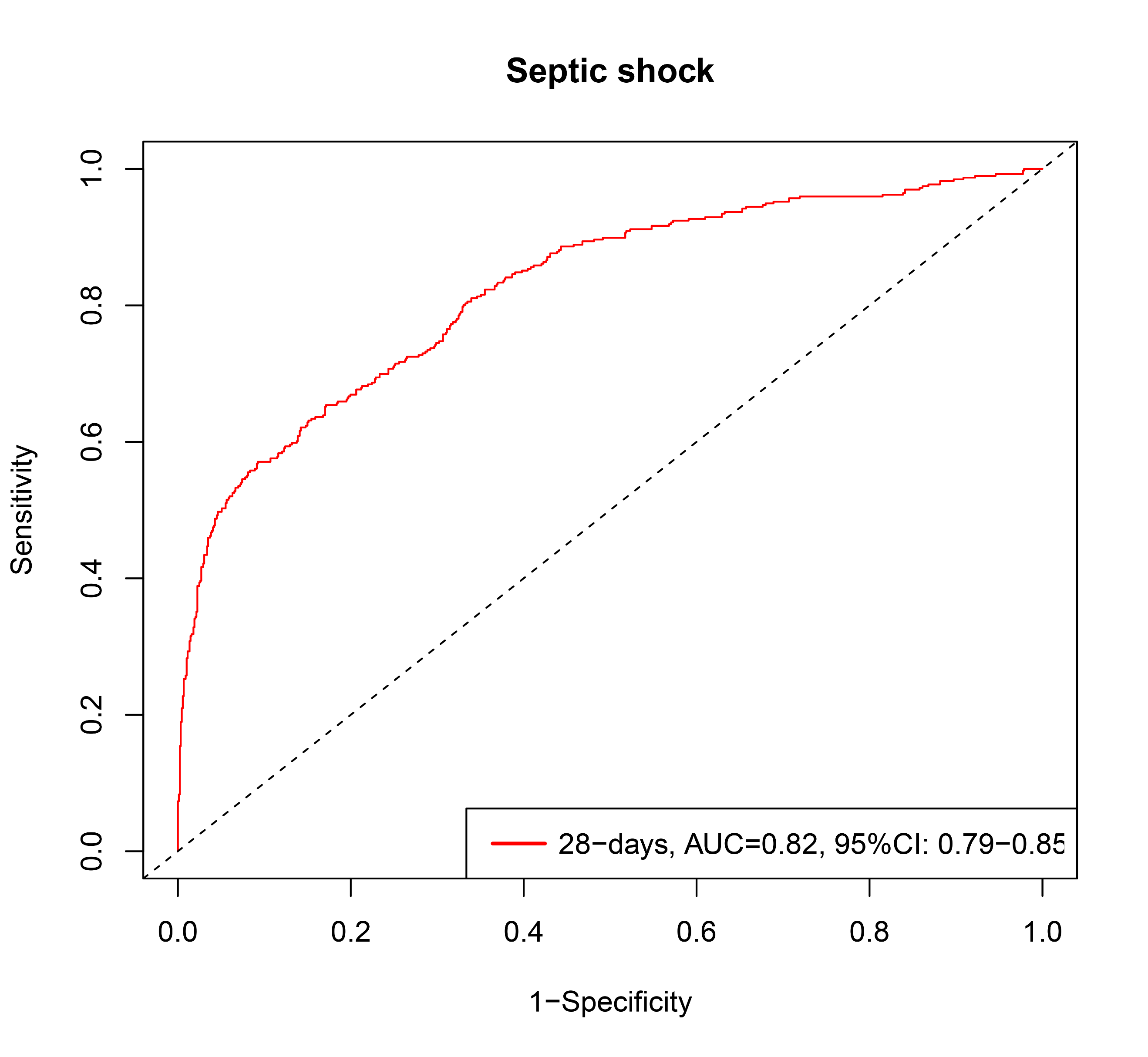


**Figure S2**. The calibration curves for evaluating the discrimination of the nomogram in septic shock patients with DM.


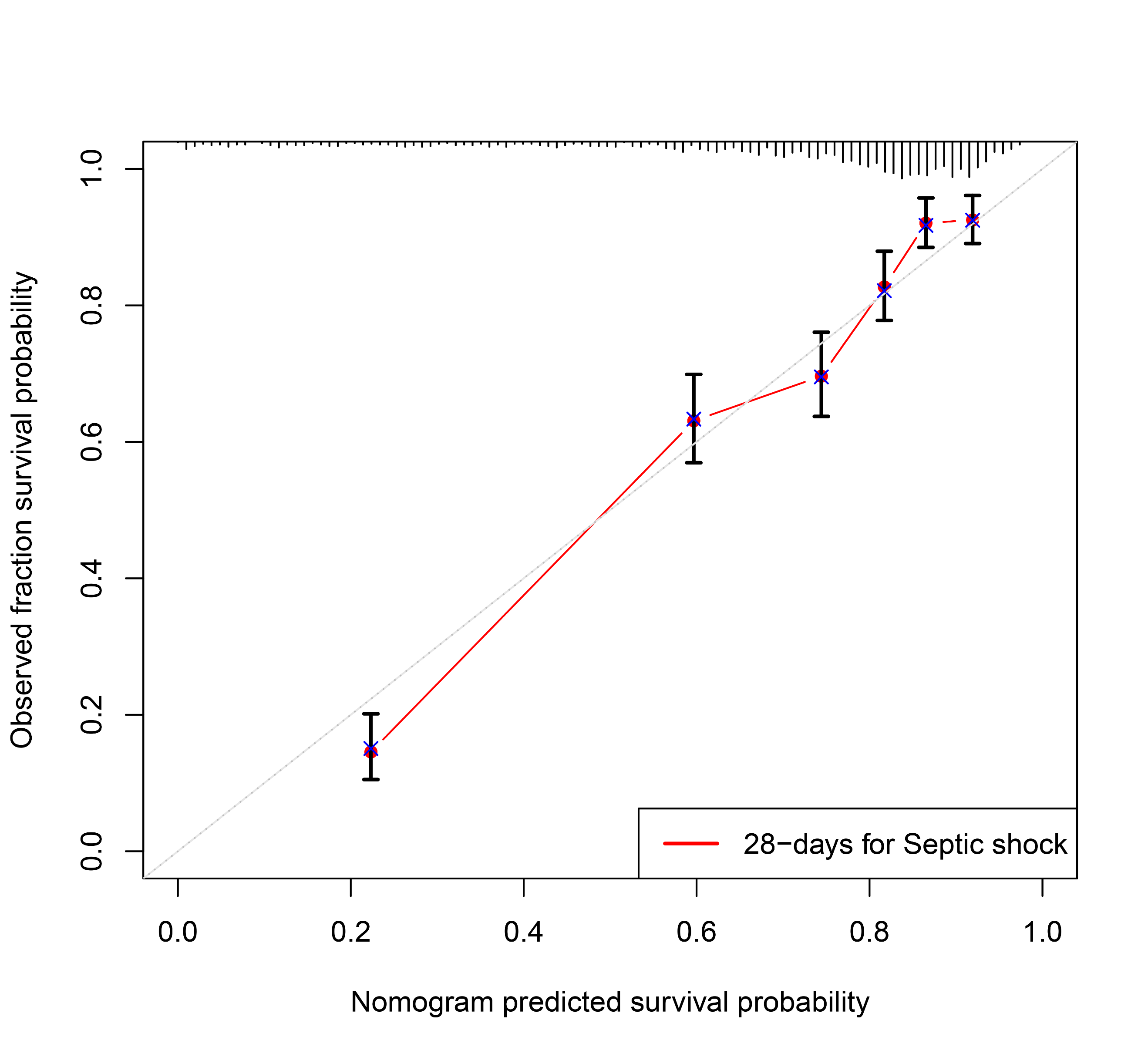


**Figure S3**. The DCA for evaluating the clinical utility of the nomogram in septic shock patients with DM.


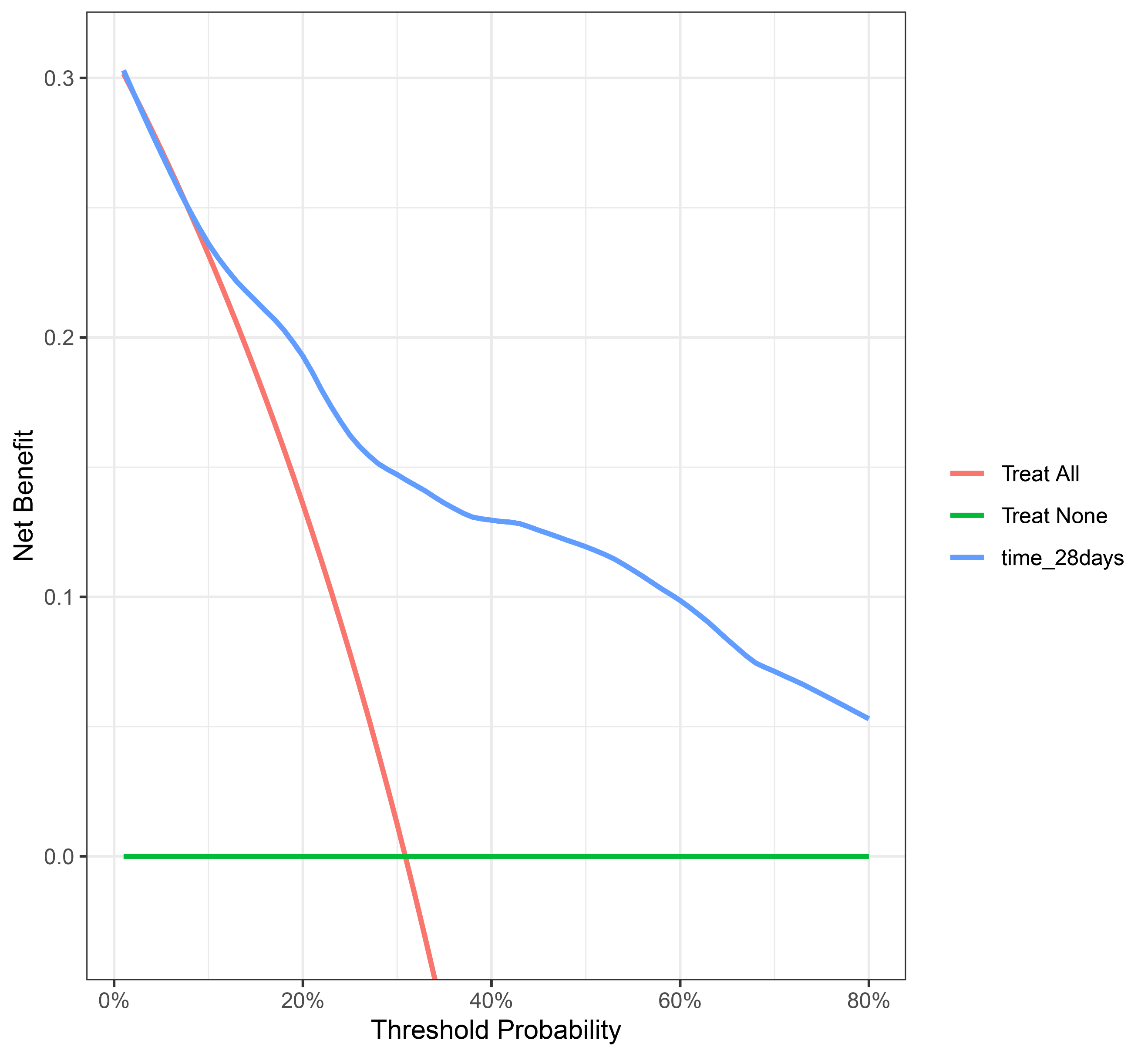

Supplement: Supplementary file 1 [file DataSheet_1.docx]
